# Altered expression of Arabidopsis genes in response to a multifunctional geminivirus pathogenicity protein

**DOI:** 10.1186/s12870-014-0302-7

**Published:** 2014-11-18

**Authors:** Lu Liu, Ho Yong Chung, Gabriela Lacatus, Surendranath Baliji, Jianhua Ruan, Garry Sunter

**Affiliations:** Department of Computer Science, The University of Texas at San Antonio, One UTSA Circle, San Antonio, TX USA; Department of Biology, The University of Texas at San Antonio, One UTSA Circle, San Antonio, TX USA; Current address: Scripps Health/Hematology/Oncology Division, 15004 Innovation Drive, San Diego, CA 92128 USA; Current address: Bayer CropScience Vegetable Seeds, 7087 East Peltier Road, Acampo, California 95220 USA

**Keywords:** Geminiviruses, Microarray, Pathogenesis, Expression, Regulatory networks

## Abstract

**Background:**

Geminivirus AC2 is a multifunctional protein that acts as a pathogenicity factor. Transcriptional regulation by AC2 appears to be mediated through interaction with a plant specific DNA binding protein, PEAPOD2 (PPD2), that specifically binds to sequences known to mediate activation of the *CP* promoter of *Cabbage leaf curl virus* (CaLCuV) and *Tomato golden mosaic virus* (TGMV). Suppression of both basal and innate immune responses by AC2 in plants is mediated through inactivation of SnRK1.2, an Arabidopsis SNF1 related protein kinase, and adenosine kinase (ADK). An indirect promoter targeting strategy, via AC2-host dsDNA binding protein interactions, and inactivation of SnRK1.2-mediated defense responses could provide the opportunity for geminiviruses to alter host gene expression and in turn, reprogram the host to support virus infection. The goal of this study was to identify changes in the transcriptome of Arabidopsis induced by the transcription activation function of AC2 and the inactivation of SnRK1.2.

**Results:**

Using full-length and truncated AC2 proteins, microarray analyses identified 834 genes differentially expressed in response to the transcriptional regulatory function of the AC2 protein at one and two days post treatment. We also identified 499 genes differentially expressed in response to inactivation of SnRK1.2 by the AC2 protein at one and two days post treatment. Network analysis of these two sets of differentially regulated genes identified several networks consisting of between four and eight highly connected genes. Quantitative real-time PCR analysis validated the microarray expression results for 10 out of 11 genes tested.

**Conclusions:**

It is becoming increasingly apparent that geminiviruses manipulate the host in several ways to facilitate an environment conducive to infection, predominantly through the use of multifunctional proteins. Our approach of identifying networks of highly connected genes that are potentially co-regulated by geminiviruses during infection will allow us to identify novel pathways of co-regulated genes that are stimulated in response to pathogen infection in general, and virus infection in particular.

**Electronic supplementary material:**

The online version of this article (doi:10.1186/s12870-014-0302-7) contains supplementary material, which is available to authorized users.

## Background

The *Geminiviridae* family comprises a large and diverse group of viruses that infect a wide range of important monocotyledonous and dicotyledonous crop species and cause significant yield losses [1,2]. Viral pathogenesis depends on a series of interactions between virus, host and insect vector. As very few viral proteins are encoded by geminiviruses, they rely, in large part, on the replication and transcription machinery of the host. One consequence of this host dependence is that geminiviruses are useful models for providing novel insights into the control of both plant and animal DNA replication and transcription.

**Figure 1:**
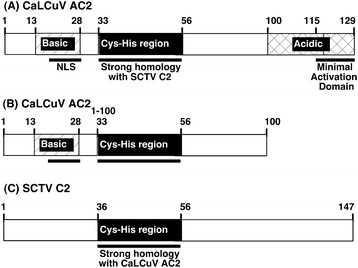
**Diagram of CaLCuV AC2 and SCTV C2 proteins used in over-expression studies. (A)** The linear drawing represents functional domains (span of amino acids indicated) present within the full-length CaLCuV AC2 protein. The N-terminal region contains a basic region of four arginine residues and a potential nuclear localization sequence. The C-terminus contains a minimal transcription activation domain within an acidic region. A region containing conserved cysteine and histidine residues forms a putative zinc finger domain, with a high degree of homology with the SCTV C2 protein. **(B)** Truncated form of the CaLCuV AC2 protein lacking the C-terminal 29 amino acids containing the acidic activation domain. **(C)** Full-length SCTV C2 protein, which lacks an acidic activation domain, but has homology to the putative zinc finger domain in CaLCuV AC2.

The circular single-stranded DNA (ssDNA) genome of geminiviruses is amplified in the nuclei of infected cells by rolling circle (RCR) and recombination-dependent (RDR) replication using cellular DNA polymerases [3,4]. The resulting double-stranded DNA replicative forms (RF) are used as template for generation of viral transcripts by host RNA polymerase II. Geminiviruses produce small multifunctional proteins to compensate for a limited coding capacity. For example, begomoviruses including *Cabbage leaf curl* (CaLCuV) and *Tomato golden mosaic* (TGMV) *virus*, code for a pathogenicity protein, AC2 (Figure 1A), that modulates metabolism [5,6], regulates transcription [7,8] and suppresses RNA silencing [9-11].

AC2 (also known as AL2 and TrAP) is required for expression of the coat protein (*CP*) and BR1 movement protein genes of both CaLCuV and TGMV [12-15]. It has been shown that AC2 is capable of inducing *CP* expression through two distinct and independent mechanisms. In mesophyll cells AC2 activates the *CP* promoter, but in vascular tissue AC2 acts to derepress the promoter [7,12]. Distinct sequences mediate activation and derepression by AC2. Sequences required for activation are located within the common region upstream of the *CP* transcription start site [8,12], whereas sequences required for repression are located 1.2 to 1.5 kbp upstream of CP transcription start site [7,12]. Among begomoviruses, the transcription function of AC2 is not virus specific as both CaLCuV or TGMV AC2 proteins can transactivate the TGMV coat protein (*CP*) promoter [12,16].

AC2 does not appear to be a canonical transcription factor as it does not bind dsDNA efficiently and appears to be targeted to responsive promoters via protein-protein interactions with cellular factors. A recent study has identified a plant specific DNA binding protein, PEAPOD2 (PPD2), that specifically binds to sequences known to mediate activation of the *CP* promoter of CaLCuV and TGMV in mesophyll cells [17]. If AC2 is targeted to responsive promoters via protein:protein interactions, we would predict that these interactions will in turn lead to activation of host genes important for pathogenesis. An indirect promoter targeting strategy, via AC2-host dsDNA binding protein interactions, might provide the opportunity for geminiviruses to alter host gene expression and in turn, reprogram the host to support virus infection. One finding that supports this idea is that AC2 can transactivate *CP* promoter-reporter transgenes integrated into cellular chromosomes [7,12], indicating that AC2 can gain access to the host chromosome.

The transcription function of AC2 is dependent on the C-terminal 29 amino acids [18], which contains an acidic activation domain (Figure 1A). AC2 also exhibits transcription-independent functions involving interactions with different cellular proteins involved in RNA silencing suppression and modulation of metabolism, mediated through sequences lacking the activation domain (Figure 1B). The L2/C2 homolog of curtoviruses (Figure 1C), including *Beet curly top* (BCTV) and *Spinach curly top* (SCTV) *virus,* share limited sequence homology with CaLCuV AC2 and lack any semblance of a transcriptional activation domain [19]. Despite the limited homology, curtovirus C2 protein does suppress RNA silencing and modulate metabolism, but does not regulate transcription [16]. The TGMV AC2, BCTV C2 and SCTV C2 proteins have been shown to interact with SnRK1.2; an Arabidopsis SNF1 related protein kinase (AKIN11) [5,19]. The consequence of this interaction is inhibition of kinase activity. Expression of an antisense SnRK1.2 transgene in *Nicotiana benthamiana* plants leads to increased susceptibility to infection [5]. The SnRK1 protein kinases play an important role in regulating energy balance in eukayotes and are members of a conserved family of protein kinases [5].

Related to this interaction, AC2 and C2 [6,19,20] also interact with and inactivate adenosine kinase (ADK). Evidence that adenosine kinase activity is reduced in virus-infected tissue and in transgenic plants expressing AC2/C2 [6,20], and that ADK-deficient plants display silencing defects [21], supports a link between silencing suppression by AC2/C2, ADK and methylation. Recent evidence indicates that the silencing suppression activity of geminivirus AC2/C2 proteins is a consequence of ADK inactivation. This is supported by results demonstrating that the ability of these proteins to suppress transcriptional gene silencing is accomplished by inhibition of ADK, which results in interference with methylation [22].

A link between ADK and SnRK1.2 is provided by evidence that SnRK1 kinases are known to be activated upon binding of 5′-AMP [23], and ADK phosphorylates adenosine producing 5′-AMP [6]. Thus, AC2 and C2 may interact with and inactivate both SnRK1.2 and ADK to prevent SnRK1-mediated metabolic (stress) responses that could enhance resistance to geminivirus infection [5]. This underscores the importance of SnRK1-mediated responses to host defense, but exactly how suppression of these responses leads to suppression of host defenses, specifically the consequence for host gene expression, has not been examined. The complex interactions and functions of geminivirus AC2 in regulating transcription and suppressing host defense mechanisms warrants the need to further investigate the host genes that respond to geminivirus AC2 protein during an infection.

Some microarray profiling of genome-wide changes in the transcriptome in response to geminivirus infection has been performed [24]. However, the asynchronous nature of an infection causes significant difficulties in determining host genes responsive to a single viral gene product. To overcome these difficulties we chose to analyze global changes in gene expression in response to the effects of a single gene, AC2. A previous study has been performed using *Mungbean yellow mosaic virus* and *African cassava mosaic virus* AC2 proteins [25]. In these studies, RNA profiling was performed in Arabidopsis protoplasts and so we chose to use a whole plant infusion assay for Arabidopsis [26]*.* The focus of this study was to identify changes in host gene expression induced by the transcription-dependent function of the viral AC2 protein, and induced by the interaction of AC2 with SnRK1. We identified large-scale changes in host gene expression in both cases. Further, computational analysis identified potential regulatory networks that respond to the two functions of AC2. Lastly, we validated the response of the top hits within these networks.

## Results and discussion

### Expression profiling of CaLCuV AC2, AC2_1-100_, SCTV C2 and asSnRK1.2 in infiltrated Arabidopsis plants

For these experiments we used full length and truncated versions of the *AC2* gene from CaLCuV, and the full-length *C2* gene from SCTV (Figure 1), as both viruses are known to cause an infection in Arabidopsis. SnRK1.2 is an endogenous Arabidopsis gene, which interacts with both AC2 and C2, and expression of antisense (as) SnRK1.2 increases the susceptibility of plants to infection [5]. We monitored the expression of CaLCuV AC2, AC2_1-100,_ SCTV C2, asSnRK1.2 and an empty plasmid vector control (pMON530) over three days to determine the time at which RNA capable of expressing each gene could be detected. Total RNA was isolated from whole Arabidopsis plants at one to three days post-infusion (dpi) with *Agrobacterium* cultures containing each DNA. Transcription directed by each construct was confirmed by RT-PCR analysis and resulting cDNA products subjected to DNA gel blot hybridization analysis using specific probes. In all cases specific cDNA products of the predicted size were detected in samples at one, two and three days, post-infusion (data not shown). As it was expected that protein and subsequent changes in host gene expression would be detectable at these time points, we used RNA isolated one and two days dpi. In addition, at these time points no phenotypic effects were observed in the *Arabidopsis* plants. Thus, these time points could be more representative of early events rather than late time points where a phenotype, such as senescence, represents the end of a signaling response. For the microarray analysis, Arabidopsis plants were vacuum infiltrated with *Agrobacterium* capable of expressing each of the constructs along with a vector control (pMON530) to eliminate effects due to *Agrobacterium* infection. Total RNA was isolated from four individual plants, one and two dpi, for three independent sets of plants infused with the different constructs. This results in three independent samples per treatment per time point. Total RNA from the samples was converted into cRNA, hybridized to the Arabidopsis ATH1 Genome Array, processed and scanned in parallel. Raw intensity data was pre-processed and normalized using the Robust Multi-array Average (RMA) procedure in MATLAB Bioinformatics Toolbox. Differentially expressed genes between experimental samples and controls were detected using two-sample t-tests with a p-value of 0.05 as the cutoff. Overall, the variability of the assay is within reasonable range and expected. The average Pearson correlation coefficient (PCC) between biological replicates is 0.971 and the average PCC between the vector controls is slightly smaller, 0.956.

### Differential expression of genes responding to CaLCuV AC2

**Figure 2:**
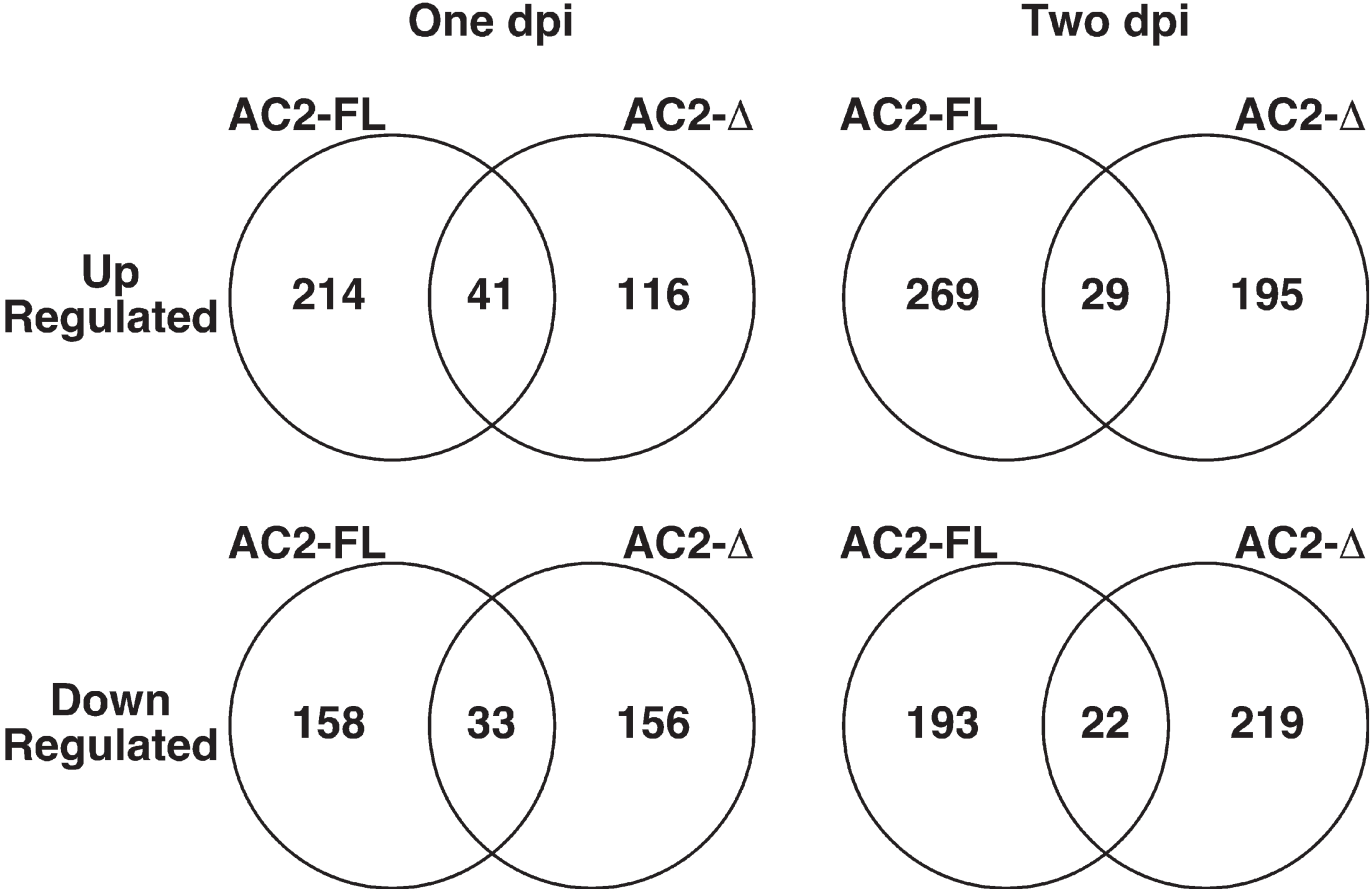
**Numbers of genes differentially expressed in response to geminivirus pathogenicity factors.** Venn diagrams illustrating the intersection between up- and down-regulated genes in Arabidopsis leaves expressing full-length (FL) or truncated (Δ) versions of CaLCuV AC2 for one and two dpi respectively.

One of the main goals of this study was to identify genes that are differentially expressed in response to the trancriptional activation function of AC2. To do this we compared the transcriptome in Arabidopsis leaves expressing full-length AC2 (FL) or a truncated AC2 (DEL), lacking the C-terminal 29 amino acids containing the acidic activation domain (AC2_1-100_) at one and two dpi (Additional file 1: Table S1 and Additional file 2: Table S2). We observed 214 genes that were specifically up-regulated by full length AC2 protein at one dpi and 269 at two dpi (Figure 2). For genes that were down-regulated, a total of 158 genes specifically responded to full length AC2 protein at one dpi, and 193 at two dpi. As the difference between the two proteins is the presence of the C-terminal activation domain in the full length protein we conclude that these potentially represent genes differentially regulated in response to the transcription function of AC2.

In samples over expressing a truncated AC2 protein we detected 116 and 195 genes specifically up-regulated at one dpi and two dpi respectively. For genes specifically down regulated by the truncated AC2 protein, 156 were detected at one dpi and 219 at two dpi. Given that the truncated AC2 protein lacks the C-terminal activation domain, we conclude that these may represent genes differentially regulated in response to the known interactions of AC2 with the cellular proteins SnRK1.2 and/or ADK [5,6]. It is of course possible that there are additional, hitherto unknown, functions within the AC2 protein that could result in differential gene expression.

Interestingly, we observed that 41 and 29 genes were up-regulated in Arabidopsis leaves expressing both full length and truncated AC2 protein at one dpi and two dpi respectively. In addition, 33 and 22 genes were down-regulated in leaves expressing both full length and truncated AC2 protein at one and two dpi respectively (Figure 2). We would expect these genes to be differentially regulated in response to the interaction with SnRK1.2 and/or ADK, given that these are functions common to both full-length and truncated AC2 protein.

To further analyze the genes where expression was differentially regulated in response to the transcription function of AC2, we made a comparison to microarray data from Arabidopsis plants infected with CaLCuV [24]. We observed a number of genes in our study that were also detected during CaLCuV infection (Additional file 3: Table S3). Of the genes up-regulated by full-length AC2 and CaLCuV-infection at two dpi, several that had functions related to RNA metabolism, including a DEA(D/H)-box RNA helicase (At3g58510) and Argonaute 2 (AGO2) (At1g31280). It is interesting that AGO2, which binds viral siRNAs and regulates innate immunity against viral infection, is up-regulated in response to AC2 and that AC2 suppresses RNA silencing. We also detected an RNA-dependent RNA polymerse gene (RdRp) (At2g19930), which functions in amplification of the RNA silencing signal, that was down-regulated in response to both AC2 and CaLCuV-infection at one dpi. Thus, it is possible that AC2 acts as an effector that is recognized by the plant, activating the innate immune response, and then acts to overcome RNA silencing. The number of genes shared between both experimental data sets were realtively small and no statistical significance was measured. However, we observed that the number of genes shared between the two data sets increased three to four-fold at two dpi (Additional file 3: Table S3). Differences observed between the two experimental data sets may be reflective of the different time scales being used in each experiment. The profiling study for CaLCuV was performed at 12 days post infection, in comparison to this study where profiling was performed one and two days after infusion. In addition this study used agroinfiltration where AC2 would be expressed in all cell types, in comparison to a systemic infection where a small number of phloem cells actually contain virus [24]. Despite this, the observation that some AC2-responsive genes are differentially regulated during virus infection, gives added confidence that we are analyzing genes relavant to viral infection.

### Functional categorization of genes differentially regulated in response to the transcription function of CaLCuV AC2

We have focused our analysis on those genes that were differentially regulated specifically in response to full-length AC2. This is interpreted to represent, at least in part, those genes differentially regulated in response to the transcriptional activation domain of full length AC2 protein. To categorize these genes by biological process we used the DAVID Bioinformatics Resource (http://david.abcc.ncifcrf.gov/summary.jsp). Most of the GO biological process categories were represented among the significant genes, but several categories were significantly enriched as compared to the Arabidopsis genome as a whole. Specifically, genes in the categories of DNA/RNA Metabolism, Transcription, Response to Stress, Protein Metabolism, Signal transduction, Cell organization and Biogenesis, Transport and Electron transport or Energy pathways were enriched at day one and day two (Additional file 4: Table S4 and Additional file 5: Table S5 respectively).

### Network analysis of genes differentially regulated in response to full length AC2

To allow us to more specifically focus on genes co-regulated in response to the transcription function of the AC2 protein we performed a network analysis. To this end, we overlayed these genes to a whole-genome co-expression network derived from more than 1000 Arabidopsis Affymetrix microarray experiments, where two genes are connected by an edge if their expression levels are highly correlated across all experimental conditions (see Methods). Our previous results showed that the connections between genes indeed suggest functional associations, and that the whole network contains many relatively independent, densely connected, sub-networks that contain co-regulated functional gene modules [27]. Interestingly, while most of the full length AC2-specific genes do not have direct connections to other AC2 responsive genes, indicating that AC2 regulates diverse functional processes, a small fraction of them are tightly linked to each other, resulting in dense sub-networks that may represent the core functional modules regulated by the transcription function of full length AC2.

**Figure 3:**
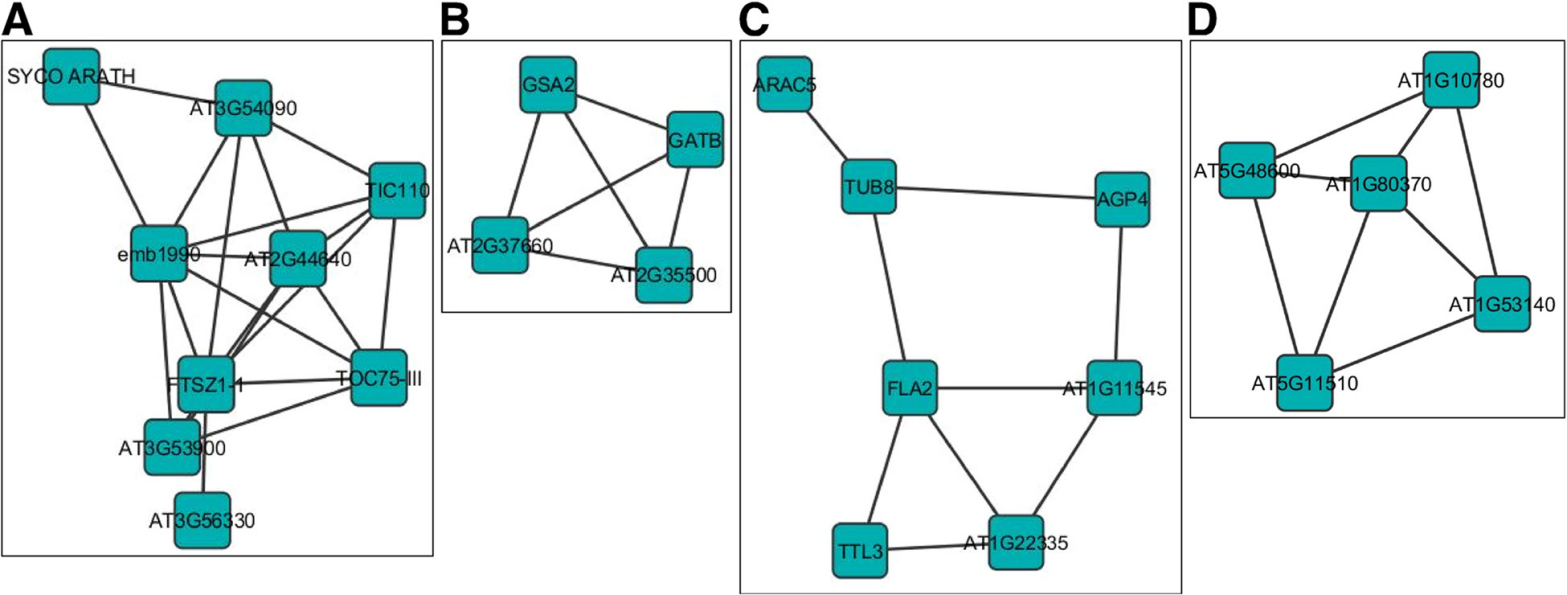
**Sub-networks of genes up-regulated in the Arabidopsis genome in response to full-length CaLCuV AC2 protein.** The diagrams illustrate sub-networks of genes that may be co-regulated in Arabidopsis, in response to the transcription activation domain of AC2. Sub-networks I **(A)**, V **(B)** and IV **(C)** were up-regulated at one dpi. Highly linked genes in sub-network IV **(D)** were up-regulated at two dpi. The sub-networks were selected from the network analysis presented in (Additional file 6: Figure S1).

Of the 214 unique genes that were up regulated in response to full length AC2 at one dpi, five sub-networks consisting of between four and eight highly connected genes were identified (Additional file 6: Figure S1A). Within these, it is interesting to note that two sub-networks (Additional file 6: Figure S1A; I and V) contained genes having functions associated with the chloroplast (Figure 3A, B). Alterations of the chloroplast transcriptome may be of interest to geminivirus infections given that chloroplasts contain components of the salicylic acid and jasmonic acid biosynthetic pathways, which elicit defense responses to viral and bacterial pathogens [28]. For example, two highly linked genes in sub-network I, Translocon at the Inner envelope membrane of Chloroplasts 110 (TIC110) and Translocon at the Outer envelope membrane of Chloroplasts 75-III (TOC75-III), are associated with complexes involved in protein import into chloroplasts. There appears to be two systems driving protein import into the chloroplast stroma, both of which utilize heat shock proteins as the motor [29]. One system utilizes heat shock cognate 70 kDa protein (cpHSC70-1), as part of the chloroplast translocon for general import, and is of potential relevance for geminivirus infections. It has been recently determined that stromules (thin projections from plastids) containing cpHSC70-1 are induced in plants infected with *Abutilon mosaic virus* (AbMV) [30]. Alteration of plastid structures and stromule biogenesis is known to occur during viral infection, and also relevant to RNA-virus infections [30]. Thus, it has been suggested that this may be important for intra- and intercellular movement of geminiviruses, given the interaction between cpHSC70-1 and the AbMV movement protein [30]. It is also worth noting that stromule formation is strongly induced in plants responding to pathogen infection, and that chloroplast structure may undergo alterations following pathogen recognition [31].

Another sub-network (Additional file 6: Figure S1A; IV), consists of genes encoding proteins associated with the cell wall and/or cytoskeleton (Figure 3C). There has been substantial work on the involvement of cytoskeletal and membrane components on plant virus movement, with many viruses encoding proteins that interact with the cytoskeleton [32]. The possibility that viruses can utilize host membranes for movement has increased based on observations that there are numerous diverse viruses that replicate in association with membranes [32]. Geminiviruses including *Bean dwarf mosaic virus*, encode a movement protein (MP) that alters the size exclusion limit of plasmodesmata to promote movement of the viral genome to adjacent cells [33]. In contrast, the *Squash leaf curl virus* MP induces the formation of ER-derived tubules, which mediate transport of a viral protein–DNA complex to adjacent cells [34]. While the relationship of genes in these sub-networks to viral pathogenesis is currently unknown, it is interesting to speculate that AC2 may induce host genes that are important for cell-to-cell and long-distance movement of the virus. This would support the known role of AC2 in activating transcription of the BR1 nuclear shuttle protein in begomoviruses to facilitate movement of the virus [14].

Of the six sub-networks identified within the 269 genes that were up-regulated in response to full length AC2 protein at two dpi (Additional file 6: Figure S1B), one may be of particular interest. The highly linked genes within sub-network IV (Figure 3D), all appear to have functions related to the cell cycle. One gene encodes the MYB domain protein 3R-4 (At5g11510), which is a transcription factor that positively regulates cytokinesis [35]. However, activation appears to require phosphorylation of the C-terminal domain of the protein, since unphosphorylated MYB3R4 acts as a repressor of mitosis [36]. In fact, a functional MYB3R4 protein appears to be required for establishment of the endocycle, which is induced in response to powdery mildew infection [36]. This may be extremely relevant to geminiviruses, especially as ploidy increases during CaLCuV infection [24], and *Maize streak virus* RepA protein induces endoreduplication [37]. Alterations in expression of cell cycle-associated and core cell cycle genes in response to CaLCuV infection suggests specific activation of S phase and inhibition of M phase, as a possible mechanism to induce the endocycle [24]. A second gene, Cyclin A2;4 (At1g80370), also up regulated in response to full-length AC2, plays a role in determining the balance between mitosis and the endocycle. However, it has been suggested that an absence or reduction in CYCA2 levels controls endoreduplication, and that expression of CYCA2 is achieved through the protein, Increased Level of Polyploidy1 (ILP1) [38]. Interestingly, ILP1 levels were elevated in CaLCuV infected leaves, although no change in the expression of CYCA2 genes was detected [24]. In contrast, an increase in the expression of CYCA2;4 was detected in transgenic Arabidopsis plants expressing BCTV L2 [39].

**Figure 4:**
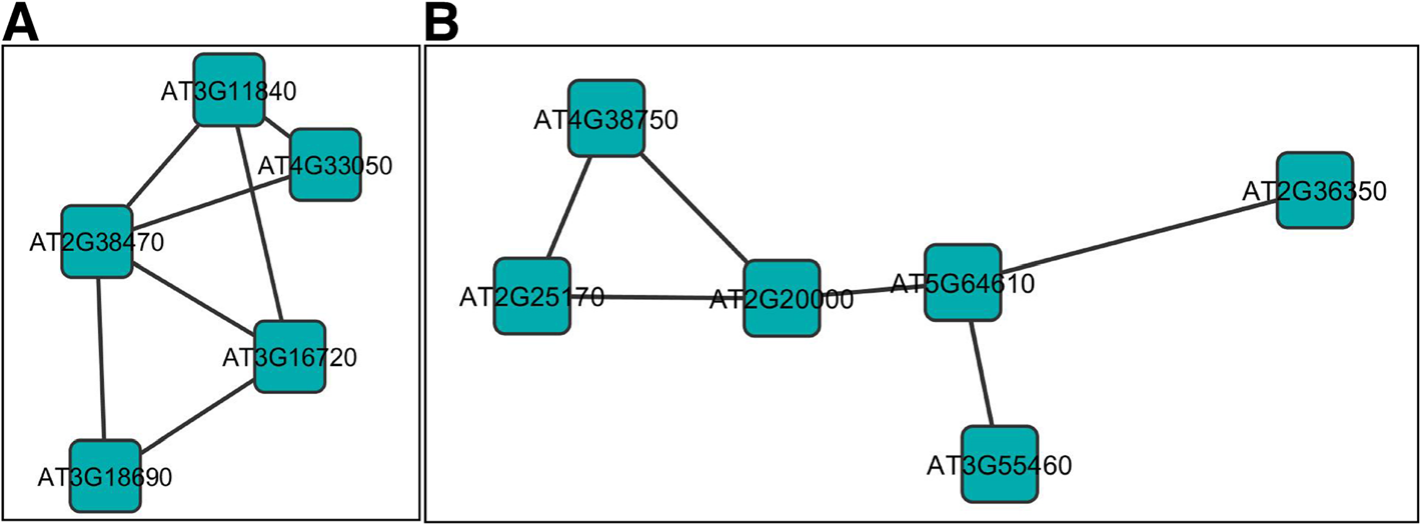
**Sub-networks of genes down-regulated in the Arabidopsis genome in response to full-length CaLCuV AC2 protein.** The diagrams illustrate sub-networks of genes that may be co-regulated in Arabidopsis, in response to the transcription activation domain of AC2. Genes within sub-network I **(A)** and sub-network IV **(B)** were down-regulated at one and two dpi respectively. The sub-networks were selected from the network analysis presented in (Additional File 7: Figure S2).

For the 158 unique genes that were down regulated in response to full length AC2 at one dpi (Additional file 7: Figure S2A), five of these were highly connected in a network of genes that are co-regulated, and all five appear to be involved in the defense response to pathogen infection (Figure 4A). MAP Kinase Substrate 1 (MKS1) is a substrate for MAP kinase 4 (MPK4), which in Arabidopsis regulates pathogen defense responses. Overexpression of MKS1 appears to be sufficient to activate SA-dependent resistance, and MKS1 interacts with WRKY transcription factors, including WRKY33, which is an *in vitro* substrate of MPK4 [40]. As different domains of MKS1 interact with MPK4 and WRKY it has been suggested that these proteins play a role in transcription or chromatin remodeling complexes, contributing to MPK4-regulated defense activation [40]. The fact that steady state mRNA levels for MKS1 and WRKY33 are down-regulated by AC2, could be interpreted as a strategy to circumvent SA-dependent responses to virus infection. Two other genes connected to MKS1 and WRKY33 are E3 ubiquitin ligases. PUB24 is a U-box-type E3 ubiquitin ligase, which acts to negatively regulate PAMP-triggered immunity (PTI) [41]. Pathogen infection leads to an increase in expression of PUB24, but decreased expression results in an impaired ability to down-regulate responses triggered by PAMPs [41]. Toxicos En Levadura 2 (ATL2), a RING-H2 Ubiquitin E3-Ligase, is rapidly induced in response to elicitors, including chitin, and may function to mediate ubiquitination of negative regulators of defense response [42]. Thus, down-regulation of this gene by AC2 would prevent degradation of proteins involved in turning off defense responses, thus preventing the host from initiating a response to infection. Interestingly, WRKY33, ATL2 and Embryo Sac Development Arrest 39 (EDA39), a calmodulin binding protein in this regulatory network, are also induced in response to chitooctaose, an elicitor of plant defense responses against pathogens [43]. Therefore, it appears as though this network of genes could be a high value target for geminiviruses.

At two dpi, 193 genes were down-regulated in response to the full length AC2 protein, and two sub-networks were detected consisting of highly connected genes (Additional file 7: Figure S2B). Within sub-network II (Figure 4B), two genes are of potential relevance for geminivirus pathogenicity. Expression of full length AC2 down-regulated cytokinin-hypersensitive 2 (CKH2; At2g25170), which encodes PICKLE, a protein similar to the CHD3 class of SWI/SNF chromatin remodeling factors [44]. Mutations within this gene result in rapidly growing green calli, which is attributed to hypersensitivity to cytokinins, where cytokinin-responsive genes respond to much lower levels of cytokinin [44]. Down regulation of CKH2 by CaLCuV AC2 could be interpreted as a mechanism to induce cytokinin responses in order to promote cell proliferation and therefore viral replication. Some evidence for this conclusion is provided by data demonstrating that begomovirus AC2, and curtovirus C2, proteins increase cytokinin-responsive promoter activity and that application of exogenous cytokinin increases susceptibility to geminivirus infection [26].

A second gene within this sub-network that is down-regulated by AC2 is Hobbit (HBT; At2g20000), which encodes a homolog of the CDC27/Nuc2/BimA/APC3 subunit of the anaphase-promoting complex (APC) [45]. The HBT protein regulates M-phase progression. *HBT* transcripts mainly accumulate around the G_2_/M phase in dividing cells, and mutations in the *HBT* gene interfere with post-embryonic cell division and differentiation of different cell types [45]. This gene may therefore be a valuable target for geminiviruses as down-regulation would presumably interfere with progression of cell differentiation shifting the balance in favor of cell proliferation, possibly in conjunction with down-regulation of CKH2 to promote cell proliferation.

### Validation of microarray results by quantitative real-time PCR

**Figure 5:**
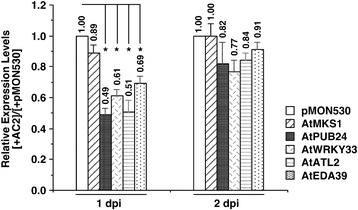
**Quantitative (q)PCR analysis of genes differentially regulated in response to full length CaLCuV AC2 protein.** Values were determined by qPCR analysis of total RNA isolated from Arabidopsis leaves infused with Agrobacterium containing DNA capable of expressing full-length *Cabbage leaf curl virus* AC2, or an empty plasmid vector (pMON530). The columns represent relative mRNA levels in CaLCuV AC2-infused leaves as compared to levels present in leaves infused with *Agrobacterium* containing empty plasmid vector (pMON530), which was arbitrarily assigned a value of 1 at each time point. The fold change was calculated from the mean ∆∆Ct values from three independent experiments using RNA isolated one and two days post-infusion (dpi). Error bars represent the Standard Error of the mean and asterisks indicate significant differences in expression as determined using the Student’s *t*-test (P < 0.05) on ∆Ct values.

For this analysis we focused on a single network that contained five down-regulated genes associated with plant defense, that were found to be highly connected at one dpi after expression of full-length AC2 (Figure 4A). Even though these five genes were only differentially regulated at one dpi in the microarray analysis, total RNA was isolated at both one and two dpi from Arabidopsis leaves infused with *Agrobacterium* containing DNA capable of expressing full-length AC2 or a vector control. After generation of cDNA, quantitative real time PCR (qPCR) analysis was performed using gene-specific primers (Additional file 8: Table S6) to verify differential regulation. As can be seen (Figure 5), at one dpi expression of AtPUB24, AtWRKY33, AtATL2 and AtEDA39 were all significantly down regulated up to two fold in samples from leaves infused with AC2 relative to samples from leaves treated with empty vector (pMON530). However, at two dpi no significant difference in expression was detectable for any of the four genes, although expression was still lower than that in samples from leaves treated with empty vector (Figure 5). These results are consistent with the microarray data, where these genes were significantly down regulated at one dpi but not at two dpi (Additional file 1: Table S1 and Additional file 2: Table S2 respectively). Interestingly, expression of AtMKS1 was not significantly altered at one dpi (Figure 5) in samples from leaves infused with AC2 relative to samples from leaves treated with empty vector (pMON530). The reasons for this are not clear but may be a consequence of differences between the two methods, including but not limited to, the utilization of vastly different normalization procedures, different strategies in probe design and sensitivity limits of PCR vs. hybridization-based approaches [46].

### Differential expression of genes responding to inactivation of SnRK1 by SCTV C2 or asSnRK1.2

**Figure 6:**
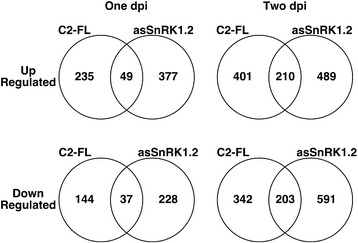
**Numbers of genes differentially expressed in response to SCTV C2 and antisense SnRK1.2.** Venn diagrams illustrating the intersection between up- and down-regulated genes in Arabidopsis leaves expressing SCTV C2 or antisense SnRK1.2, for one and two dpi respectively.

A second goal of this study was to examine the consequence(s) of the interaction between SCTV C2 and SnRK1.2. To do this we compared the transcriptomes in Arabidopsis leaves expressing full-length SCTV C2 or an antisense construct of SnRK1.2 (asSnRK1.2) at one and two dpi (Additional file 9: Table S7 and Additional file 10: Table S8). The rationale for this approach is that interaction between geminvirus AC2 and C2 proteins results in inactivation of the kinase [5,19], and asSnRK1.2 is expected to result in degradation of sense mRNA through the siRNA pathway and lead to loss of SnRK1.2 activity. Thus, genes found to be differentially regulated in response to both treatments is presumed to be a consequence of reduced SnRK1.2 activity. Of those genes up-regulated in response to C2 or asSnRK1, 49 were common to both treatments at one dpi and 210 at two dpi (Figure 6). For genes down-regulated in response to C2 or asSnRK1.2 at one or two dpi, we observed 37 and 203 respectively, that were common to both treatments (Figure 6). These genes are therefore interpreted to represent genes responding to inhibition of SnRK1 activity by geminvirus C2 protein. It is important to note here that the total number of genes differentially regulated in response to both C2 and asSnRK1 was ~ five-fold higher at day two (Figure 6).

Some differentially regulated genes were specific to each individual treatment. Of those genes specifically up-regulated by SCTV C2, we detected 235 at one dpi and 401 at two dpi (Figure 6). 144 and 342 genes were specifically down-regulated by SCTV C2, at one and two dpi respectively. Presumably, these genes are differentially regulated in response to additional functions of SCTV C2, which would include interaction with and inactivation of ADK [6], and possibly additional unknown functions. There were also many genes whose expression changed specifically in response to expression of asSnRK1.2. At day one and two dpi, we detected 377 and 489 genes respectively, up-regulated in response to asSnRK1 alone (Figure 6). For genes down-regulated in response to asSnRK1 alone, 228 and 591 were detected at one and two dpi respectively (Figure 6). As these genes were not differentially regulated in response to SCTV C2, we conclude that this may be a consequence specific to SnRK1.2 activity.

### Functional categorization of genes differentially regulated in response to asSnRK1.2

The focus of this analysis was to characterize genes found to be differentially regulated in response to both SCTV C2 and asSNRK1.2. We categorized these genes by biological process using the DAVID Bioinformatics Resource. Most of the GO biological process categories were represented among the significant genes, but several categories were significantly enriched as compared to the Arabidopsis genome as a whole. In this case, genes associated with Transcription, Protein Metabolism and Transport, and Electron transport or Energy pathways were over-represented (Additional file 11: Table S9 and Additional file 12: Table S10).

### Network analysis of genes differentially regulated in response to inactivation of SnRK1.2

**Figure 7:**
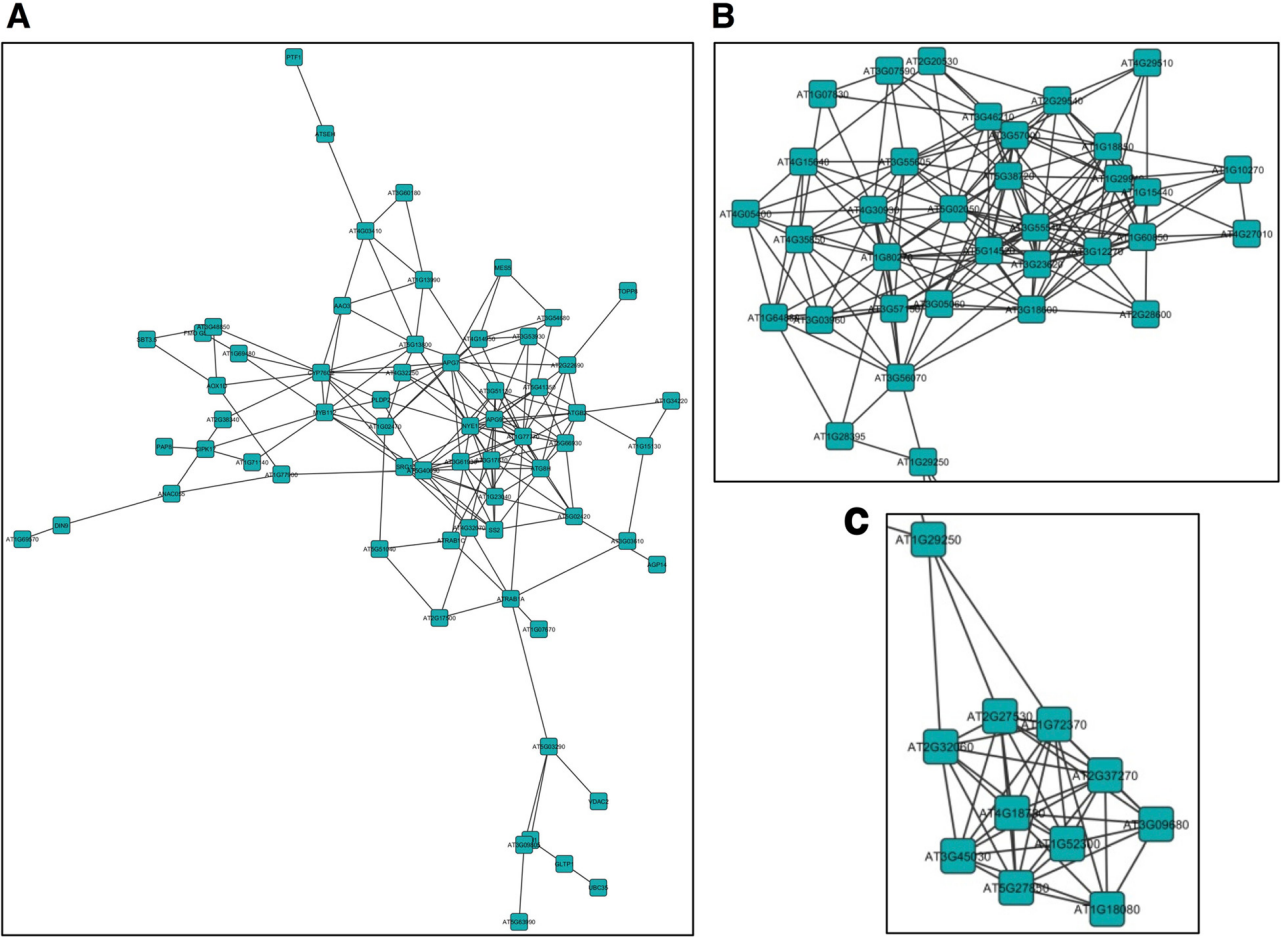
**Sub-networks of genes differentially regulated in response to full-length CaLCuV AC2 protein.** The diagrams illustrate sub-networks of genes that may be co-regulated in response to to both SCTV C2 and asSnRK1.2 at two dpi. **(A)** Network of genes up-regulated at two dpi. **(B)** and **(C)** Networks of genes down-regulated at two dpi. The sub-networks were selected from the network analysis presented in (Additional file 13: Figure S3 and Additional file 14: S4). A list of the connections between genes in the networks (edges) is given in (Additional file 15: Table S11).

We overlayed the asSnRK1.2 responsive genes to the Arabidopsis co-expression network, and extracted dense subnetworks for further investigation. Given the small number of genes that were up- (Additional file 13: Figure S3A) or down- (Additional file 14: Figure S4A) regulated in response to both SCTV C2 and asSnRK1.2 at one dpi, no networks consisting of highly connected genes were identified. However, at two dpi a large increase in the number of genes that were up- (Additional file 13: Figure S3B) and down- (Additional file 14: Figure S4B) regulated revealed complex networks (Additional file 15: Table S11). Of the 209 genes that were up regulated in response to SCTV C2 and asSnRK1.2 at two dpi, a large complex network was identified (Figure 7A), within which several genes have functions associated with autophagy. This is a process by which cytoplasmic contents, including proteins and organelles, are sequestered within the autophagosome, a double-membrane vesicle, which can deliver the contents to lysosomes or vacuoles through fusion for degradation [47]. Autophagy is involved in both the responses to biotic stresses, including viral infection, and in regulating senescence, and many autophagy genes have been identified and functionally analyzed in plants. Of the three genes within this network found to be up-regulated in response to C2 and asSnRK1.2, the role of the APG9 (At2g31260) complex is unclear. However, APG7 (At5g45900) is an E1 ubiquitin-activating enzyme that conjugates phosphatidylethanolamine to ATG8H (AT3G06420) [48]. More evidence is being provided that autophagy may function either to facilitate or prevent viral pathogenesis [49,50]. As a defense against pathogen infection, autophagy has been shown to play an important role in both pathogen-induced hypersensitive cell death (HR), and the plant antiviral immune response. Rapid immune responses, including HR, are induced in tobacco plants carrying the N-resistance gene when infected by *Tobacco mosaic virus* (TMV). The result of this is limitation on the replication and systemic spread of the virus [51]. Silencing of BECLIN1/ATG6*,* ATG3, or APG7 resulted in the spread of cell death, suggesting that autophagy plays an anti-death role during pathogen infection to limit the spread of HR beyond initially infected cells [52]. A suppressor of programmed cell death in tomato (Adi3) has been shown to interact with tomato ATG8H although it is not clear at this time whether Adi3 is targeted by autophagy [53]. Since autophagy is an emerging antiviral process employed by the host immune system, certain viruses have successfully evolved to either avoid, subvert or even actively induce autophagy to ensure a productive infection [54]. Interestingly, autophagy-related transcripts, including ATG8H and ATG9, were up regulated during infection of tomato with *Tomato yellow leaf curl* Sardinia virus (TYLCSV) [55] and in Arabidopsis infected with CaLCuV [24].

Of particular relevance to geminiviruses are recent studies that have shown a role for autophagy in RNA silencing [50]. This is an antiviral response that results in dsRNA-mediated degradation of viral RNAs. As a counter-defense, viruses encode RNA silencing suppressors (RSSs) that act to suppress the RNA silencing machinery [9]. A recent study indicates that a tobacco regulator of gene silencing calmodulin-like protein (Nt-rgsCaM) binds to an arginine-rich region within a number of viral RSSs, resulting in degradation through autophagosomes [56]. This supports the idea that autophagy can provide a secondary antiviral mechanism by targeting viral RSSs for degradation. However, we have recently demonstrated that in the case of geminiviruses, there appears to be a different mechanism where AC2, the begomovirus RSS, induces rgsCaM and may in fact sequester rgsCaM in the nucleus to prevent targeting of AC2 for degradation via the autophagy pathway [57]. While we cannot explain this apparent discrepancy, it could reflect a difference between the RNA viruses used in one study [56] and geminiviruses in our study [57]. Recently, it has been shown that the polerovirus P0 RSS targets Argonaute 1 (AGO1) for degradation via the autophagy pathway [58]. At this time it is unknown whether AC2 specifically targets genes in the autophagy pathway to facilitate pathogenesis.

Of further interest to geminivirus pathogenesis is the observation that under conditions of stress, including pathogen infection, AMPK appears to regulate the autophagy pathway through two mechanisms. First, AMPK directly interacts with Ulk1, an autophagy initiator, through phosphorylation [59]. AMPK can indirectly induce autophagy through phosphorylation of raptor, which inhibits the mTORC1 complex [60]. Thus, phosphorylation of Ulk1 by mTORC1 and/or AMPK results in either negative or positive regulation of autophagy respectively [61]. The geminvirus AC2/C2 proteins have been shown to interact with and inactivate SnRK1, the plant homolog of AMPK [5]. Under the stress of viral infection, this would prevent phosphorylation of raptor maintaining an active mTORC1 complex. This would ensure that the autophagy pathway is inhibited. Secondly, inhibition of SnRK1 by AC2/C2 would prevent direct phosphorylation of Ulk1, again preventing activation of the authophagy pathway. However, there is an apparent paradox given that we detect up-regulation of autophagy genes in response to both full length SCTV C2 and asSnRK1.2. This can be partially explained by observations that the autophagosome marker ATG8 is rapidly up regulated under starvation conditions in yeast, and that most of the autophagy genes are regulated at a transcriptional level [62]. This reiterates the importance of SnRK1 as a high value target for geminiviruses [5,6,20,26], by preventing activation of autophagy in the event of up-regulation of genes in that pathway.

For the 203 common genes that were down regulated at two dpi, a large complex network containing highly connected genes that appear to be co-regulated was identified (Additional file 14: Figure S4B). Two smaller clusters of genes within this network (Figure 7B and C) have functions associated with the ribosome and translation. Although the genes identified have not been specifically reported to play roles in viral pathogenesis, there are examples of ribosomal proteins that play a role in antiviral defense, and so it may not be surprising that geminiviruses down-regulate these genes to facilitate infection. With respect to geminiviruses, the nuclear shuttle protein (BR1) has been shown to target the NSP-interacting kinases (NIKs), which are leucine-rich-repeat (LRR) receptor-like-kinases (RLKs) involved in antiviral defense [63]. NIK1 phosphorylates the ribosomal protein, rpL10A, which functions as an immediate downstream effector of the NIK1-mediated response and binding of NSP to NIK1 inhibits its kinase activity preventing the antiviral defense pathway from impacting geminvirus infection [63,64].

### Validation of microarray data by quantitative real-time RT-PCR

**Figure 8:**
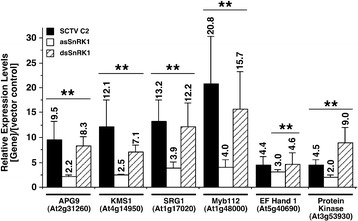
**Quantitative (q)PCR analysis of genes differentially regulated in response to inactivation of SnRK1.** Values were determined by qPCR analysis of total RNA isolated from Arabidopsis leaves infused with Agrobacterium containing DNA capable of expressing full-length *Spinach curly top virus* C2, antisense (as)SnRK1.2, an inverted repeat construct designed to express dsRNA (dsSnRK1.2) or an empty plasmid vector (pMON530). The columns represent relative mRNA levels in C2, asSnRK1, or dsSnRK1-infused leaves as compared to levels present in leaves infused with *Agrobacterium* containing empty plasmid vector (pMON530), which was arbitrarily assigned a value of 1 at each time point. The fold change was calculated from the mean ∆∆Ct values from three independent experiments using RNA isolated two days post-infusion (dpi). Error bars represent the Standard Error of the mean and asterisks indicate significant differences in expression as determined using the Student’s *t*-test (P < 0.05) on ∆Ct values.

We chose to analyze six genes with functions associated with autophagy and senescence (Figure 7A) that were up-regulated in response to both C2 and asSnRK1.2. Total RNA was isolated at both one and two dpi from Arabidopsis leaves infused with *Agrobacterium* containing DNA capable of expressing full-length C2, asSnRK1.2 or the vector control (pMON530). In addition, we also used an inverted repeat construct designed to express dsRNA (dsSnRK1.2) that is known to reduce target mRNA levels in infused *N.benthamiana* leaves [20]. After generation of cDNA, qPCR analysis was performed using gene-specific primers (Additional file 8: Table S6) to verify differential regulation. As shown (Figure 8), significant increases in expression were observed in response to SCTV C2, asSnRK1.2 and dsSnRK1.2 at two dpi for all six genes tested. No significant changes in expression were detectable at one dpi (data not shown). This is consistent with the microarray data where expression of these genes increased in response to both SCTV C2 and asSnRK1.2 (Additional file 10: Table S8). Given that we also observed up-regulation of these genes in response to silencing of SnRK1.2 with an inverted repeat construct (dsSnRK1) we interpret this to be a consequence of the inactivation/inhibition of SnRK1.2.

## Conclusion

It is becoming increasingly apparent that geminiviruses manipulate the host in several ways to facilitate an environment conducive to infection, predominantly through the use of multifunctional proteins. As one example, TGMV AL1 protein is necessary for origin recognition and initation of RCR [65,66]. TGMV AL1 also binds to a plant retinoblastoma (pRb) protein [67,68], and is sufficient for PCNA accumulation [69]. This is analogous to small DNA tumor viruses, where adenovirus and SV40 deregulate the cell cycle via interaction with the pRb and p53 pathways [70-73]. In addition, infection by CaLCuV has been shown to influence the host transcriptome [24], again demonstrating the ability of geminiviruses to manupulate the host to ensure efficient infection. A second multifunctional protein encoded by geminviruses that influences the host response to infection, is the AC2/C2 protein. We have recently shown that the CaLCuV *CP* promoter is regulated by AC2 through an interaction with PPD2, a plant specific DNA binding protein, that specifically binds sequences known to mediate activation of the *CP* promoter of CaLCuV and TGMV [17]. An indirect promoter targeting mechanism could provide an opportunity for the virus (via AC2) to alter host gene expression. This may in turn reprogram the host to support virus infection and/or evade host defense responses. Additional interactions between AC2/C2 and SnRK1.2 and ADK lead to suppression of host defenses [5,6], which could also lead to alterations in host transcriptome. In support of this, our study along with others using either whole virus infections [24] or over-expression of AC2 from ACMV or MMYMV [25,74], identified large scale changes in the host transcriptome. The other studies were performed either in whole Arabidopsis plants [24], transient assays using Arabidopsis protoplasts [25] or transgenic *Nictotiana tabacum* constitutively expressing AC2 [74]. The complexity of possible effects of AC2 makes it desirable to extend this type of analysis under different conditions to identify key host factors independent of laboratories and host plant-virus interactions. Thus, the current study is complementary to the others and provides completely novel aspects for the functional analysis. As with the other studies, we identified several categories of genes that were significantly enriched as compared to the Arabidopsis genome as a whole, including genes for DNA/RNA Metabolism, Transcription, Response to Stress, Protein Metabolism, Signal transduction, Cell organization and Biogenesis, Transport and Electron transport or Energy. Our analysis enabled us to identify networks containing highly connected genes that could reflect co-regulated functional gene modules. Two of these highlight the significance of our approach in uncovering novel clusters of genes targeted by geminiviral RSSs. As an example, sub-networks containing genes having functions associated with the chloroplast and the cell wall and/or cytoskeleton, could reflect a direct role for AC2 in inducing the expression of genes important for virus movement. The latter may have uncovered an explanation for the observation that mutations within the TGMV *AC2* gene lead to loss of infectivity [75]. This is due, primarily, to the fact that AC2 is required for the transcriptional activation of the BR1 nuclear shuttle protein which is necessary for movement of the virus [14]. Thus, alteration of genes associated with the chloroplast and cell wall and/or cytoskeleton could reflect a direct role for AC2 in inducing the expression of genes important for virus movement. It will be interesting to determine whether the promoters of the genes identified have any *cis*-acting elements in common with the *BR1* genes of begomoviruses.

In a second example, our network-based approach has identified a potential link between RNA silencing suppressors, SnRK1.2 and autophagy (Figure 7). This is supported by recent evidence demonstrating that autophagy plays a role in directing degradation of DICER and AGO2, important proteins in miRNA processing and in post-transcriptional regulation of DICER mRNA [76]. Therefore, it has been proposed that autophagy may represent a checkpoint for maintaining homeostasis of miRNA populations [76], and so it interesting to speculate that inhibition of SnRK1.2 by the geminivirus AC2/C2 proteins may have wide-reaching effects on both RNA silencing and autophagy. However, many unresolved questions remain regarding the role of autophagy in viral pathogenesis, but targeting of this pathway underscores the likely importance of autophagy as a component of antiviral immunity.

Our approach to identifying highly connected genes that are differentially regulated by AC2 has revealed co-regulated gene networks that are potentially targeted by geminiviruses during infection. Many of these genes would not have been thought of as functioning in a network, but this approach allows us to assess them as a functioning unit and determine the importance of the network as a whole in viral pathogenesis. We can now identify novel pathways of co-regulated genes that are stimulated in response to pathogen infection in general, and virus infection in particular. We are currently confirming the differential expression of genes in all the sub-networks and are investigating the role each sub-network plays in viral pathogenesis.

## Methods

### DNA constructs

Cloned DNAs capable of constitutively expressing CaLCuV AC2 (p35S-CaLCuVAC2) or SCTV C2 (p35s-SCTV C2) from the CaMV 35S promoter have been described previously [12,26] . A DNA construct capable of constitutively expressing a truncated CaLCuV AC2 protein lacking the C-terminal activation domain (CaLCuV AC2_1-100_) was generated by PCR. A 300 bp fragment was amplified with primers CaLCVAC2F (5′-gcgagatctatgcaaaattcatcactcttg-3′) and CaLCVAC2Rdel (5′-gcgctcgagctacgtaggttgtggttgaac-3′) using CaLCuV DNA A as a template. Following restriction with *Xho*I-*Bgl*II the fragment was cloned into similarly cut pMON530 to generate p35S-CaLCuVAC2_1-100_. To generate a DNA construct capable of constitutively expressing an antisense RNA to Arabidopsis SnRK1.2 (AKIN11) from the CaMV 35S promoter, pAS2-AKIN11 DNA [19] was restricted with *Nco*I and treated with Klenow to generate a blunt end. Following restriction with *Bam*HI, the resulting 1.5 kbp fragment was cloned into the plant binary vector pMON530 [76] at the *Bgl*II and *Sma*I sites, to generate DNA containing the SnRK1.2 coding region in the antisense orientation (asSnRK1.2). The presence of each ORF in the correct orientation was confirmed by DNA sequencing. The resulting Ti plasmid constructs were mobilized into *Agrobacterium* strain GV3111SE by triparental mating [77] and used for agroinfiltration. As a control, vector DNA containing the CaMV 35S promoter alone (pMON530) was introduced into *Agrobacterium*.

### Agrobacterium infusion assays and RNA isolation

Vacuum infiltration of *Arabidopsis thaliana* plants with *Agrobacterium* cultures was performed essentially as described [26]. *Arabidopsis* Col-0 plants were sprinkled with water prior to infiltration and whole plants submerged in the *Agrobacterium* culture ensuring all rosette leaves were submerged in the solution. Vacuum was drawn for 20-30 min at a pressure of approximately 0.05 Bar. Plants were removed from the beaker, replanted into moist soil, covered and placed in a growth chamber under long day conditions (16 h light and 8 h dark) and incubated at 21°C. Infiltrations were performed in the afternoon and infiltrated leaf tissue from four different plants harvested in the afternoon one to three days post-inoculation, depending on the experiment. Total RNA was isolated from infiltrated leaves of Arabidopsis using Plant RNA Reagent as described by the manufacturer (Invitrogen, Carlsbad, CA), treated with DNaseI (Ambion, Austin, TX) and purified through RNeasy MiniElute clean up kit (Qiagen, Valencia, CA).

### GeneChip hybridization and microarray data analysis

Affymetrix ATH1 GeneChips (Affymetrix P/N 510690), containing more than 22,500 probe sets representing ~24,000 genes, were used through out the experiment and all procedures were carried out according to the manufacturers instructions (Affymetrix, Santa Clara, CA). For one comparison, Arabidopsis plants were infused with *Agrobacterium* cultures containing CaLCuV AC2, CaLCuV AC2_1-100_, or empty plasmid vector (pMON530). In a second comparison, Arabidopsis plants were infused with *Agrobacterium* cultures containing SCTV C2, asSnRK1.2 or empty plasmid vector (pMON530). Three independent experiments were performed for each comparison, at different times, and total RNA isolated from infused plants at one and two days post-infiltration. This resulted in a total of nine samples for each comparison at one and two dpi. Comparison 1: Nine arrays for samples 530 × 3, CaLCuV AC2 × 3, CaLCuV AC2_1-100_ × 3 at day one and two = 18 total. Comparison 2: Nine arrays for samples 530 × 3, SCTV C2 × 3, asSnRK1.2 × 3 at day one and two = 18 total. Total RNA (10 μg) was processed by a one-step labeling protocol (Affymetrix), and fragmented cRNA (15 μg) hybridized to the Arabidopsis ATH1 Genome using the recommended standard procedures (45°C for 16 h). Washing and staining were performed in a fluidics station 400, using the standard protocol EUkGEWS2v4 and scanned using an Agilent GeneArray Scanner. Array quality was assessed following the parameters recommended by Affymetrix (GeneChip Expression Analysis, Technical Manual, 701021 rev 1). Raw intensity data was processed using The Robust Multi-array Average (RMA) procedure in MATLAB Bioinformatics Toolbox, which first performs background adjustment and quantile normalization on the probe level, and then summarizes the intensity levels from each probe set to gene-level expression values in logarithmic scale [78]. Fold changes, while not used for selecting differentially expressed genes, were computed by first taking the arithmetic mean of the log2(gene expression) of the three biological replicates, and then calculating the ratio of the mean expression values in linear scale. From a total of 22810 genes represented on the array, genes differentially expressed between experimental samples and controls were detected using two-sample t-tests with a multiple-testing corrected p-value of 0.05 used as the cutoff. Permutation test with 1000 permutations was performed to correct for multiple testing [79].

There are many different methods for defining/selecting differentially expressed (DE) genes and each could result in a different set of genes. In general, fold change, while simple and intuitive, is not a preferred criterion in selecting DE genes, because of lack of indication in the level of confidence and reproducibility [78]. It is important to note that fold change is not necessarily a biologically more meaningful measure than statistical significance, as some genes can have their effects at very low level of fold changes while some other genes need to function at a much higher level. In addition, the fold change approach is usually subject to bias as it tends to select low-intensity genes whose fold change values have a larger variance than the fold change values of high-intensity genes. Last but not least, raw intensity data from microarray experiments often need to be preprocessed and normalized, which could dramatically impact the fold change estimation, depending on the procedure used, leaving the definition of fold change obscure. We choose our approach based on a study that shows, with Affymetrix arrays in particular, the t-test usually results in more accurate discovery of DE genes, especially when combined with RMA for preprocessing and normalization [80]. At the same time, the study also showed that RMA often produces a biased estimation of fold change, which is probably the reason that the observed fold changes for the DE genes in our experiment are relatively small. Simulations in their study showed that RMA can reduce the fold change by as much as 2 fold (e.g. a 4-fold change could be reduced to 2-fold after RMA).

### Statistical and network-based analysis

Over-representation of Gene Ontology (GO) terms within each gene list was performed using the hypogeometric test implemented on the DAVID Bioinformatics Resource [81]. To identify sub-networks for a list of genes, we overlayed these input genes to an Arabidopsis gene co-expression network [27] using gene expression data from >1300 microarray experiments, and retrieved sub-networks that consists of only the input genes and their connections. For genes down-regulated at two dpi by asSnRK1.2, as the returned network is very large, we iteratively removed genes with less than four connections in the sub-network and the remaining sub-network is used for further analysis.

### Quantitative real-time PCR

Quantitative real-time PCR (qPCR) was used to assess differences in the steady state mRNA levels of genes in response to the proteins of interest by comparison to a plasmid vector treated control. Total RNA (1 μg) isolated from Arabidopsis leaf tissue was treated with DNase I and reverse transcribed using a high-capacity cDNA archive kit (Applied Biosystems, Foster city, CA). qPCR analysis was performed with SYBR Green using gene specific probes (Additional file 8: Table S6), with a 7500 Real-time PCR system (Applied Biosystems, Foster city, CA) as described previously [26], or with the Biomark HD System (Fluidigm Corporation). Primer sequences were designed using Primer Express 2.0 software (Applied Biosystems). For each experiment, target samples were normalized to EF1α, which was used as an reference. In each experiment, samples from three independent biological samples were used for the analysis. Ct values for each well position were examined prior to data analysis. Differences in gene expression (ΔΔCt) were calculated using the 7500 System SDS software package (Applied Biosystems, Foster city, CA), which measured differences in expression of the target gene and the endogenous control (ΔCt) in each replicate.

### Availability of supporting data

The microarray dataset used in this manuscript has been deposited with the Gene Expression Omnibus (GEO) and assigned the following GEO accession number: GSE62180. All of the data can be accessed through the following link: http://www.ncbi.nlm.nih.gov/geo/query/acc.cgi?acc=GSE62180.

## Additional files

Additional file 1: Table S1. Genes differentially expressed in response to CaLCuV AC2. Genes that are up- or down regulated at one dpi, in response to full-length AC2 (FL) or a deletion derivative lacking the transcription activation domain (DEL), are shown. Additional file 2: Table S2. Genes differentially expressed in response to CaLCuV AC2. A list of genes that are up- or down-regulated at two dpi, in response to a full-length AC2 (FL) or a deletion derivative lacking the transcription activation domain (DEL), are shown. Additional file 3: Table S3. A comparison of differentially regulated genes found in CaLCuV-infected Arabidopsis with those found in response to full-length (FL) AC2. The first two worksheets list genes up- or down-regulated in Arabidopsis plants infected with CaLCuV at 12 days post infection [24] and indicates whether the same genes were also differentially regulated in response to FL AC2. A hypergeometric distribution is shown in the third worksheet. Additional file 4: Table S4. GO biological process categories represented among the genes differentially expressed in response to CaLCuV AC2 at one dpi. Additional file 5: Table S5. GO biological process categories represented among the genes differentially expressed in response to CaLCuV AC2 at two dpi. Additional file 6: Figure S1. Network analysis using genes that were up-regulated specifically in response to full length AC2. Sub-networks (red boxes) containing highly connected genes that were up-regulated in response to full length AC2 at one **(A)** or two **(B)** dpi. Additional file 7: Figure S2. Network analysis using genes that were down-regulated specifically in response to full length AC2. Sub-networks (red boxes) containing highly connected genes that were up-regulated in response to full length AC2 at one **(A)** or two **(B)** dpi. Additional file 8: Table S6. Primer sets used for qPCR analysis in this study. Additional file 9: Table S7. Genes differentially expressed in response to inactivation of Arabidopsis SnRK1.2. Genes that are up- or down-regulated at one dpi, in response to SCTV C2 or antisense SnRK1.2, are shown. Additional file 10: Table S8. Genes differentially expressed in response to inactivation of Arabidopsis SnRK1.2. Genes that are up- or down-regulated at two dpi, in response to SCTV C2 or antisense SnRK1.2, are shown. Additional file 11: Table S9. GO biological process categories represented among the genes differentially expressed in response to inactivation of Arabidopsis SnRK1.2 at one dpi. Additional file 12: Table S10. GO biological process categories represented among the genes differentially expressed in response to inactivation of Arabidopsis SnRK1.2 at two dpi. Additional file 13: Figure S3. Network analysis using genes that were up-regulated specifically in response to full length AC2. Sub-networks (red boxes) containing highly connected genes that were up-regulated in response to SCTV C2 and antisense SnRK1.2 at one **(A)** or two **(B)** dpi. Additional file 14: Figure S4. Network analysis using genes that were down-regulated specifically in response to full length AC2. Sub-networks (red boxes) containing highly connected genes that were up-regulated in response to SCTV C2 and antisense SnRK1.2 at one **(A)** or two **(B)** dpi. Additional file 15: Table S11. Network Edges: A list of connections between two genes in the networks shown in Figure 7.

## Abbreviations

35S: CaMV promoter
5′-AMP: 5′ adenosine monophosphateAbMV, *Abutilon mosaic virus*
AC2: Begomovirus transcriptional activator protein
ADK: Adenosine kinase
AKIN11: Arabidopsis SNF1 related protein kinase 1.2
asSnRK1.2: Antisense version ofArabidopsis SnRK1.2
Atg: autophagy related genes
ATL2: RING-H2 Ubiquitin E3-Ligase
BCTV: *Beet curly top virus*
BR1: Begomovirus nuclear shuttle protein
CaLCuV: *Cabbage leaf curl virus*
CaMV: *Cauliflower mosaic virus*
CP: Coat protein
cpHSC70-1: Chloroplast heat shock cognate 70 kDa protein
cRNA: Complementary RNA
dpi: Days post-infusion
DNA: Deoxyribonucleic acid
dsDNA: Double stranded DNA
EDA39: Embryo Sac Development Arrest 39
EF1α: Eukaryotic elongation factor 1α
eIF: Eukaryotic initiation factor
ER: endoplasmic reticulum
GO: gene ontology
HBT: Hobbit
ILP1: *Increased level of polyploidy1*
L2/C2: Curtovirus pathogenicity protein
LRR: Leucine rich repeat
MKS1: MAP kinase substrate 1
MP: Movement protein
MPK4: MAP kinase 4
MYB3R4: MYB domain protein 3R-4
NIK: NSP-interacting kinases
NSP: Nuclear shuttle protein
p53: Tumor suppressor protein
PAMP: Pathogen associated molecular pattern
PCNA: Proliferating cell nuclear antigen
PCR: Polymerase chain reaction
PPD2: Arabidopsis peapod2 protein
pRb: Plant retinoblastoma protein
PTI: PAMP-triggered immunity
PUB24: U-box-type E3 ubiquitin ligase
qPCR: Quantitative real time PCR
RCR: Rolling circle replication
RDR: Recombination dependent replication
RF: Replicative form
RLK: LRR receptor-like kinase
RMA: Robust multi-array averagerpL, ribosomal protein L
RNA: Ribonucleic acid
RSS: RNA silencing suppressor
RT-PCR: Reverse transcriptase PCR
SCTV: *Spinach curly top virus*
SNF1: Sucrose non-fermenting 1 protein
SnRK1: SNF1 related protein kinase
ssDNA: Single stranded DNA
SV40: *Simian virus 40*
TGMV: *Tomato golden mosaic virus*
TIC110: Translocon at the inner envelope membrane of chloroplasts 110
TMV: *Tobacco mosaic virus*
TOC75-III: Translocon at the outer envelope membrane of chloroplasts 75-III
WRKY33: Plant-specific transcription factor

## References

[1] Varma A, Malathi VG (2003). Emerging geminivirus problems: a serious threat to crop production. Annals Appl Biol.

[2] Navas-Castillo J, Fiallo-Olive E, Sanchez-Campos S (2011). Emerging virus diseases transmitted by whiteflies. Ann Rev Phytopathol.

[3] Preiss W, Jeske H (2003). Multitasking in replication is common among geminiviruses. J Virol.

[4] Stenger DC, Revington GN, Stevenson MC, Bisaro DM (1991). Replicational release of geminivirus genomes from tandemly repeated copies: evidence for rolling circle replication of a plant viral DNA. Proc Natl Acad Sci U S A.

[5] Hao L, Wang H, Sunter G, Bisaro DM (2003). Geminivirus AL2 and L2 proteins interact with and inactivate SNF1 kinase. Plant Cell.

[6] Wang H, Hao L, Shung C-Y, Sunter G, Bisaro DM (2003). Adenosine kinase is inactivated by geminivirus AL2 and L2 proteins. Plant Cell.

[7] Sunter G, Bisaro DM (1997). Regulation of a geminivirus coat protein promoter by AL2 protein (TrAP): evidence for activation and derepression mechanisms. Virology.

[8] Sunter G, Bisaro DM (2003). Identification of a minimal sequence required for activation of the *Tomato golden mosaic virus* coat protein promoter in protoplasts. Virology.

[9] Voinnet O, Pinto YM, Baulcombe DC (1999). Suppression of gene silencing: a general strategy used by diverse DNA and RNA viruses of plants. Proc Natl Acad Sci U S A.

[10] Carrington JC, Kasschau KD, Johansen LK (2001). Activation and suppression of RNA silencing by plant viruses. Virology.

[11] van Wezel R, Liu H, Tien P, Stanley J, Hong Y (2002). Mutation of three cysteine residues in *Tomato yellow leaf curl virus*-China C2 protein causes dysfunction in pathogenesis and posttranscriptional gene silencing-suppression. Mol Plant Microbe Interact.

[12] Lacatus G, Sunter G (2008). Functional analysis of bipartite begomovirus coat protein promoter sequences. Virology.

[13] Sunter G, Bisaro DM (1991). Transactivation in a geminivirus: AL2 gene product is needed for coat protein expression. Virology.

[14] Sunter G, Bisaro DM (1992). Transactivation of geminivirus AR1 and BR1 gene expression by the viral AL2 gene product occurs at the level of transcription. Plant Cell.

[15] Berger MR, Sunter G (2013). Identification of sequences required for AL2-mediated activation of the *Tomato golden mosaic virus-yellow vein* BR1 promoter. J Gen Virol.

[16] Sunter G, Stenger DC, Bisaro DM (1994). Heterologous complementation by geminivirus AL2 and AL3 genes. Virology.

[17] Lacatus G, Sunter G (2009). The *Arabidopsis* PEAPOD2 transcription factor interacts with geminivirus AL2 protein and the coat protein promoter. Virology.

[18] Hartitz MD, Sunter G, Bisaro DM (1999). The geminivirus transactivator (TrAP) is a zinc-binding phosphoprotein with an acidic activation domain. Virology.

[19] Baliji S, Sunter J, Sunter G (2007). Transcriptional analysis of complementary sense genes in *Spinach curly top virus* and the functional role of C2 in pathogenesis. Mol Plant Microbe Interact.

[20] Wang H, Buckley KJ, Yang X, Buchmann RC, Bisaro DM (2005). Adenosine kinase inhibition and suppression of RNA silencing by geminivirus AL2 and L2 proteins. J Virol.

[21] Moffatt BA, Stevens YY, Allen MS, Snider JD, Pereira LA, Todorova MI, Summers PS, Weretilnyk EA, Martin-McCaffrey L, Wagner C (2002). Adenosine kinase deficiency is associated with developmental abnormalities and reduced transmethylation. Plant Physiol.

[22] Buchmann RC, Asad S, Wolf JN, Mohannath G, Bisaro DM (2009). Geminivirus AL2 and L2 proteins suppress transcriptional gene silencing and cause genome-wide reductions in cytosine methylation. J Virol.

[23] Sugden C, Crawford RM, Halford NG, Hardie DG (1999). Regulation of spinach SNF1-related (SnRK1) kinases by protein kinases and phosphatases is associated with phosphorylation of the T loop and is regulated by 5’-AMP. Plant J.

[24] Ascencio-Ibañez JT, Sozzani R, Lee T-J, Chu T-M, Wolfinger RD, Cellab R, Hanley-Bowdoin L (2008). Global analysis of *Arabidopsis* gene expression uncovers a complex array of changes impacting pathogen response and cell cycle during geminivirus infection. Plant Physiol.

[25] Trinks D, Rajeswaran R, Shivaprasad PV, Akbergenov R, Oakeley EJ, Veluthambi K, Hohn T, Pooggin MM (2005). Suppression of RNA silencing by a geminivirus nuclear protein, AC2, correlates with transactivation of host genes. J Virol.

[26] Baliji S, Lacatus G, Sunter G (2010). The interaction between geminivirus pathogenicity proteins and adenosine kinase leads to increased expression of primary cytokinin responsive genes. Virology.

[27] Ruan J, Perez J, Hernandez B, Lei C, Sunter G, Sponsel V (2011). Systematic identification of functional modules and *cis*-regulatory elements in *Arabidopsis thaliana*. BMC Bioinformatics.

[28] Jones AME, Thomas V, Bennett MH, Mansfield J, Grant M (2006). Modifications to the *Arabidopsis* defense proteome occur prior to significant transcriptional change in response to inoculation with *Pseudomonas syringae*. Plant Physiol.

[29] Su P-H, Li H-M (2010). Stromal Hsp70 is important for protein translocation into pea and *Arabidopsis* chloroplasts. Plant Cell.

[30] Krenz B, Jeske H, Kleinow T (2012). The induction of stromule formation by a plant DNA-virus in epidermal leaf tissues suggests a novel intra- and intercellular macromolecular trafficking route. Front Plant Sci.

[31] Caplan JL, Mamillapalli P, Burch-Smith TM, Czymmek K, Dinesh-Kumar SP (2008). Chloroplastic protein NRIP1 mediates innate immune receptor recognition of a viral effector. Cell.

[32] Schoelz JE, Harries PA, Nelson RS (2011). Intracellular transport of plant viruses: finding the door out of the cell. Mol Plant.

[33] Noueiry AO, Lucas WJ, Gilbertson RL (1994). Two proteins of a plant DNA virus coordinate nuclear and plasmodesmal transport. Cell.

[34] Lazarowitz SG, Beachy RN (1999). Viral movement proteins as probes for intracellular and intercellular trafficking in plants. Plant Cell.

[35] Haga N, Kato K, Murase M, Araki S, Kubo M, Demura T, Suzuki K, Müller I, Voß U, Jürgens G, Ito M (2007). R1R2R3-Myb proteins positively regulate cytokinesis through activation of KNOLLE transcription in *Arabidopsis thaliana*. Development.

[36] Chandran D, Inada N, Hather G, Kleindt CK, Wildermuth MC (2010). Laser microdissection of *Arabidopsis* cells at the powdery mildew infection site reveals site-specific processes and regulators. Proc Natl Acad Sci U S A.

[37] Desvoyes B, Ramirez-Parra E, Xie Q, Chua NH, Gutierrez C (2006). Cell type-specific role of the retinoblastoma/E2F pathway during *Arabidopsis* leaf development. Plant Physiol.

[38] Yoshizumi T, Tsumoto Y, Takiguchi T, Nagata N, Yamamoto YY, Kawashima M, Ichikawa T, Nakazawa M, Yamamoto N, Matsuia M (2006). INCREASED LEVEL OF POLYPLOIDY1, a conserved repressor of CYCLINA2 transcription, controls endoreduplication in *Arabidopsis*. Plant Cell.

[39] Caracuel Z, Lozano-Durán R, Huguet S, Arroyo-Mateos M, Rodríguez-Negrete EA, Bejarano ER (2012). C2 from *Beet curly top virus* promotes a cell environment suitable for efficient replication of geminiviruses, providing a novel mechanism of viral synergism. New Phytologist.

[40] Andreasson E, Jenkins T, Brodersen P, Thorgrimsen S, Petersen NHT, Zhu S, Qiu J-L, Micheelsen P, Rocher A, Petersen M, Newman M-A, Bjorn Nielsen H, Hirt H, Somssich I, Mattsson O, Mundy J (2005). The MAP kinase substrate MKS1 is a regulator of plant defense responses. EMBO J.

[41] Trujillo M, Ichimura K, Casais C, Shirasu K (2008). Negative regulation of PAMP-triggered immunity by an E3 ubiquitin ligase triplet in arabidopsis. Curr Biology.

[42] Serrano M, Guzmán P (2004). Isolation and gene expression analysis of *Arabidopsis thaliana* mutants with constitutive expression of ATL2, an early elicitor-response RING-H2 Ziznc-finger gene. Genetics.

[43] Libault M, Wan J, Czechowski T, Udvardi M, Stacey G (2007). Identification of 118 Arabidopsis transcription factor and 30 ubiquitin-ligase genes responding to chitin, a plant-defense elicitor. Mol Plant Microbe Interact.

[44] Furuta K, Kubo M, Sano K, Demura T, Fukuda H, Liu Y-G, Shibata D, Kakimoto T (2011). The CKH2/PKL chromatin remodeling factor negatively regulates cytokinin responses in *Arabidopsis* calli. Plant Cell Physiol.

[45] Blilou I, Frugier F, Folmer S, Serralbo O, Willemsen V, Wolkenfelt H, Eloy NB, Ferreira PCG, Weisbeek P, Scheres B (2002). The *Arabidopsis* HOBBIT gene encodes a CDC27 homolog that links the plant cell cycle to progression of cell differentiation. Genes Develop.

[46] Wang Y, Barbacioru C, Hyland F, Xiao W, Hunkapiller KL, Blake J, Chan F, Gonzalez C, Zhang L, Samaha RR (2006). Large scale real-time PCR validation on gene expression measurements from two commercial long-oligonucleotide microarrays. BMC Genomics.

[47] Klionsky DJ (2005). The molecular machinery of autophagy: unanswered questions. J Cell Sci.

[48] Kim SH, Kwon C, Lee JH, Chung T (2012). Genes for plant autophagy: functions and interactions. Mol Cells.

[49] Shoji-Kawata S, Levine B (2009). Autophagy, antiviral immunity, and viral countermeasures. Biochim Biophys Acta.

[50] Zhou J, Yu J-Q, Chen Z (2014). The perplexing role of autophagy in plant innate immune responses. Mol Plant Pathol.

[51] Marathe R, Anandalakshmi R, Liu Y, Dinesh-Kumar SP (2002). The *Tobacco mosaic virus* resistance gene, N. Mol Plant Pathol.

[52] Liu Y, Schiff M, Czymmek K, Tallóczy Z, Levine B, Dinesh-Kumar SP (2005). Autophagy regulates programmed cell death during the plant innate immune response. Cell.

[53] Devarenne TP (2011). The plant cell death suppressor Adi3 interacts with the autophagic protein Atg8h. Biochem Biophys Res Commun.

[54] Kim HJ, Lee S, Jung JU (2010). When autophagy meets viruses: a double-edged sword with functions in defense and offense. Semin Immunopathol.

[55] Miozzi L, Napoli C, Sardo L, Accotto GP (2014). Transcriptomics of the interaction between the monopartite phloem-limited geminivirus *Tomato yellow leaf curl Sardinia virus* and *Solanum lycopersicum* highlights a role for plant hormones, autophagy and plant immune system fine tuning during infection. PLoS One.

[56] Nakahara KS, Masuta C, Yamada S, Shimura H, Kashihara Y, Wada TS, Meguro A, Goto K, Tadamura K, Sueda K, Sekiguchi T, Shao J, Itchoda N, Matsumura T, Igarashi M, Ito K, Carthew RW, Uyeda I (2012). Tobacco calmodulin-like protein provides secondary defense by binding to and directing degradation of virus RNA silencing suppressors. Proc Natl Acad Sci U S A.

[57] Chung HY, Lacatus G, Sunter G (2014). Geminivirus AL2 protein induces expression of, and interacts with, a calmodulin-like gene, an endogenous regulator of gene silencing. Virology.

[58] Derrien B, Baumberger N, Schepetilnikov M, Viotti C, De Cillia J, Ziegler-Graff V, Isono E, Schumacher K, Genschik P (2012). Degradation of the antiviral component ARGONAUTE1 by the autophagy pathway. Proc Natl Acad Sci U S A.

[59] Kim J, Kundu M, Viollet B, Guan KL (2011). AMPK and mTOR regulate autophagy through direct phosphorylation of Ulk1. Nature Cell Bio.

[60] Gwinn DM, Shackelford DB, Egan DF, Mihaylova MM, Mery A, Vasquez DS, Turk BE, Shaw RJ (2008). AMPK phosphorylation of raptor mediates a metabolic checkpoint. Mol Cell.

[61] Alers S, Loffler AS, Wesselborg S, Stork B (2012). Role of AMPK-mTOR-Ulk1/2 in the regulation of autophagy: cross talk, shortcuts, and feedbacks. Mol Cell Biol.

[62] He C, Klionsky DJ (2009). Regulation mechanisms and signaling pathways of autophagy. Ann Rev Gen.

[63] Fontes EPB, Santos AA, Luz DF, Waclawovsky AJ, Chory J (2004). The geminivirus NSP acts as virulence factor to suppress an innate transmembrane receptor kinase-mediated defense signaling. Genes Develop.

[64] Carvalho CM, Santos AA, Pires SR, Rocha CS, Saraiva DI, Machado JPB, Mattos EC, Fietto LG, Fontes EPB (2008). Regulated nuclear trafficking of rpL10A mediated by NIK1 represents a defense strategy of plant cells against virus. PLoS Pathog.

[65] Fontes EPB, Eagle PA, Sipe PS, Luckow VA, Hanley-Bowdoin L (1994). Interaction between a geminivirus replication protein and origin DNA is essential for viral replication. J Biol Chem.

[66] Orozco BM, Hanley-Bowdoin L (1996). A DNA structure is required for geminivirus origin function. J Virol.

[67] Ach RA, Durfee T, Miller AB, Taranto P, Hanley-Bowdoin L, Zambriski PC, Gruissem W (1997). An alternatively-spliced, multigene family in maize encodes retinoblastoma-related proteins which can interact with a plant D-type cyclin and a geminivirus replication protein. Mol Cell Biol.

[68] Settlage SB, Miller AB, Gruissem W, Hanley-Bowdoin L (2001). Dual interaction of a geminivirus replication accessory protein and a plant cell cycle regulator. Virology.

[69] Nagar S, Pedersen TJ, Carrick K, Hanley-Bowdoin L, Robertson D (1995). A geminivirus induces expression of host DNA synthesis protein in terminally differentiated plant cells. Plant Cell.

[70] Bargonetti J, Reynisdottir I, Friedman PN, Prives C (1992). Site-specific binding of wild-type p53 to cellular DNA is inhibited by SV40 T antigen and mutant p53. Genes Dev.

[71] de Stanchina E, McCurrach ME, Zindy F, Shieh S-Y, Ferbeyre G, Samuelson AV, Prives C, Roussel MF, Sherr CJ, Lowe SW (1998). E1A signaling to p53 involves the p19ARF tumor suppressor. Genes Dev.

[72] Dobbelstein M, Arthur AK, Dehde S, van Zee K, Dickmanns A, Fanning E (1992). Intracistronic complementation reveals a new function of SV40 T antigen that co-operates with Rb and p53 binding to stimulate DNA synthesis in quiescent cells. Oncogene.

[73] Whyte P, Buchkovich KJ, Horowitz JM, Friend SH, Raybuck M, Weinberg RA, Harlow E (1988). Association between an oncogene and an antioncogene: the adenovirus E1A proteins bind to the retinoblastoma gene product. Nature.

[74] Soitamo AJ, Jada B, Lehto K (2012). Expression of geminiviral AC2 RNA silencing suppressor changes sugar and jasmonate responsive gene expression in transgenic tobacco plants. BMC Plant Biol.

[75] Sunter G, Hartitz MD, Hormuzdi SG, Brough CL, Bisaro DM (1990). Genetic analysis of *Tomato golden mosaic virus*. ORF AL2 is required for coat protein accumulation while ORF AL3 is necessary for efficient DNA replication. Virology.

[76] Gibbings D, Mostowy S, Jay F, Schwab Y, Cossart P, Voinnet O (2012). Selective autophagy degrades DICER and AGO2 and regulates miRNA activity. Nat Cell Biol.

[77] Rogers SG, Klee HJ, Horsch RB, Fraley RT (1987). Improved vectors for plant transformation: expression cassette vectors and new selectable markers. Meth Enzymol.

[78] Irizarry RA, Bolstad BM, Collin F, Cope LM, Hobbs B, Speed TP (2003). Summaries of affymetrix GeneChip probe level data. Nuc Acids Res.

[79] Dudoit S, Yang Y, Matthew J, Speed TP (2002). Statistical methods for identifying differentially expressed genes in replicated cDNA microarray experiments. Statistica Sinica.

[80] Cui X, Churchill GA (2003). Statistical tests for differential expression in cDNA microarray experiments. Genome Biol.

[81] Huang DW, Sherman BT, Lempicki RA (2009). Systematic and integrative analysis of large gene lists using DAVID Bioinformatics Resources. Nature Protoc.

