# Supplementary figures and images for: Altered expression of Arabidopsis genes in response to a multifunctional geminivirus pathogenicity protein
Source: BMC Plant Biol. 2014 Nov 18;14:302. doi: 10.1186/s12870-014-0302-7 (PMC4253603; doi:10.1186/s12870-014-0302-7)

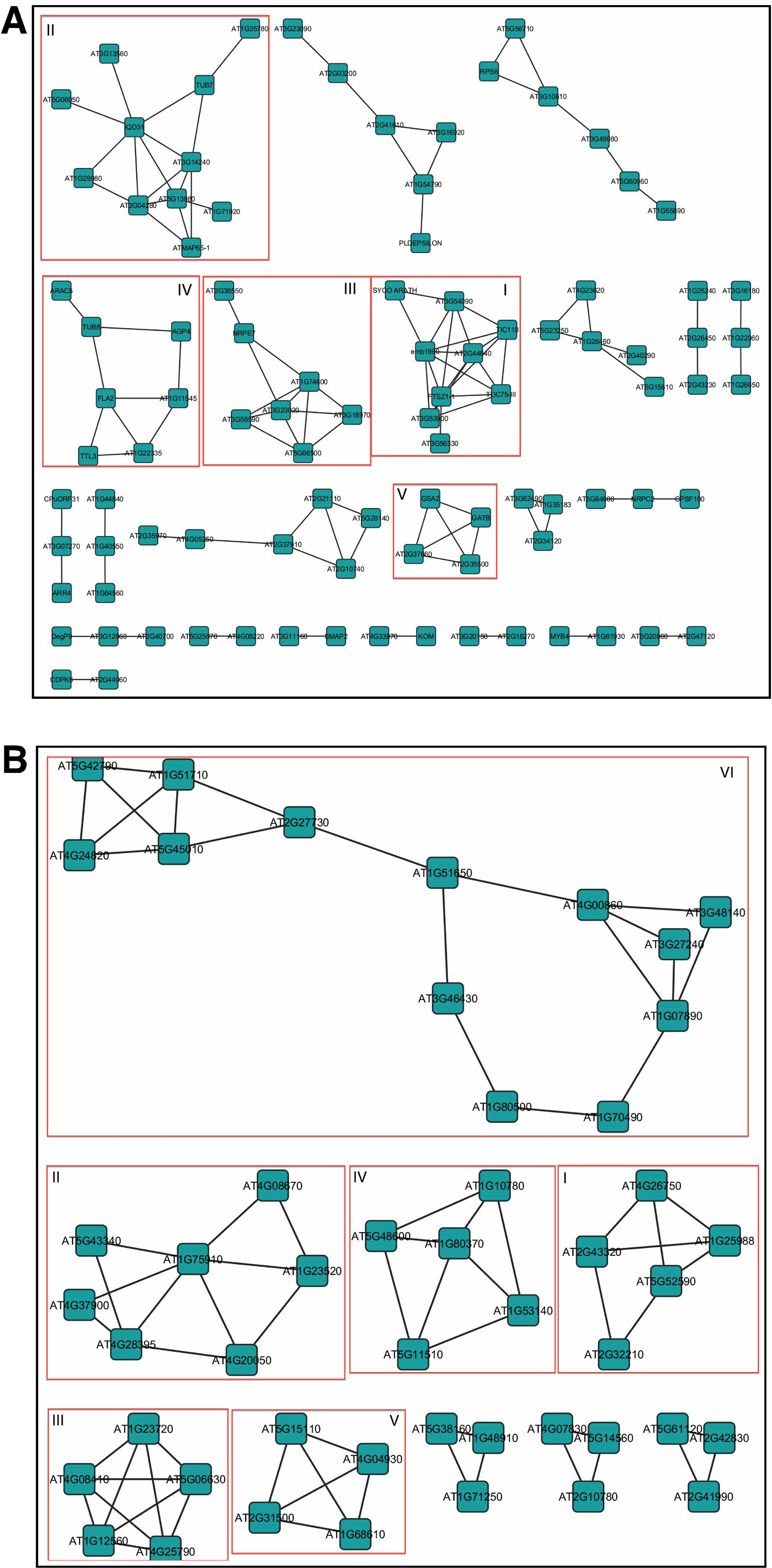

Supplement: Additional file 6: Figure S1. — Network analysis using genes that were up-regulated specifically in response to full length AC2. Sub-networks (red boxes) containing highly connected genes that were up-regulated in response to full length AC2 at one (A) or two (B) dpi. [file 12870_2014_302_MOESM6_ESM.tiff]

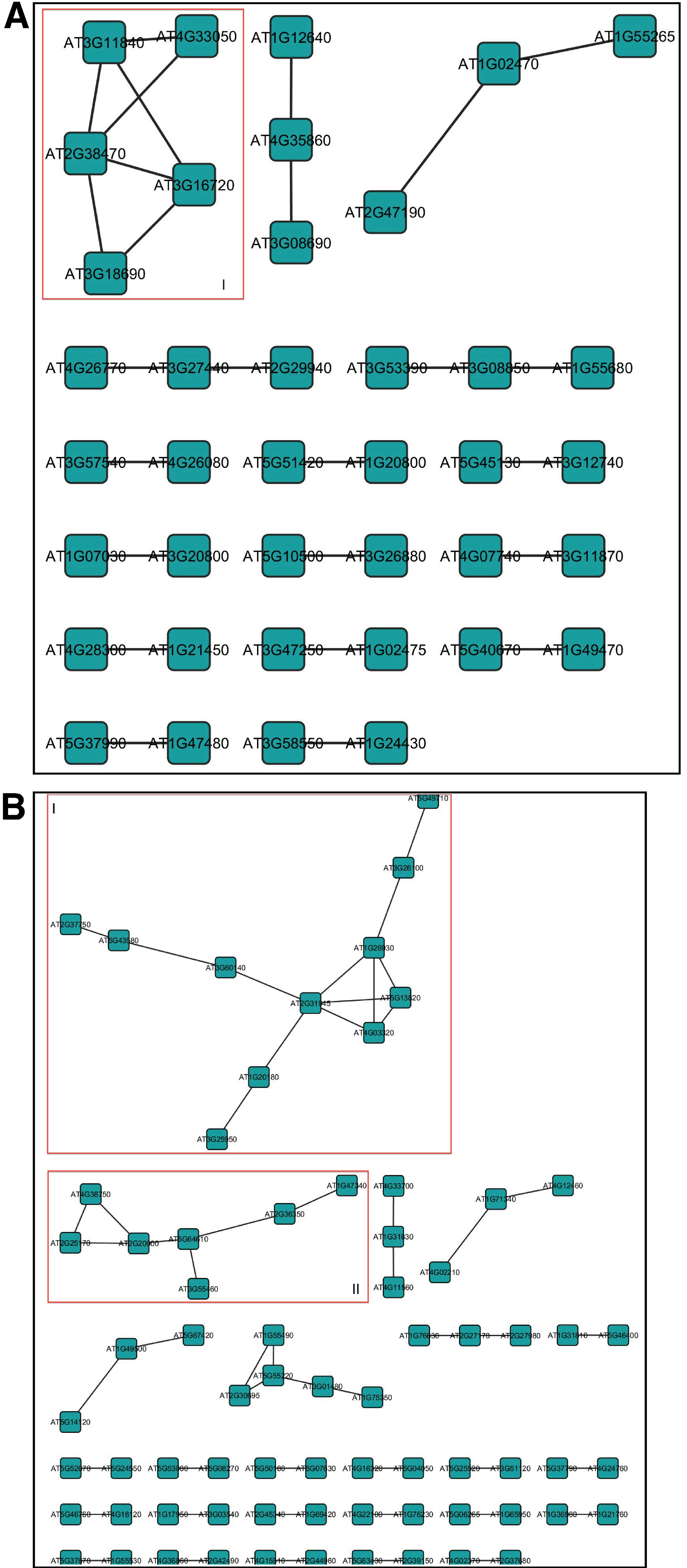

Supplement: Additional file 7: Figure S2. — Network analysis using genes that were down-regulated specifically in response to full length AC2. Sub-networks (red boxes) containing highly connected genes that were up-regulated in response to full length AC2 at one (A) or two (B) dpi. [file 12870_2014_302_MOESM7_ESM.tiff]

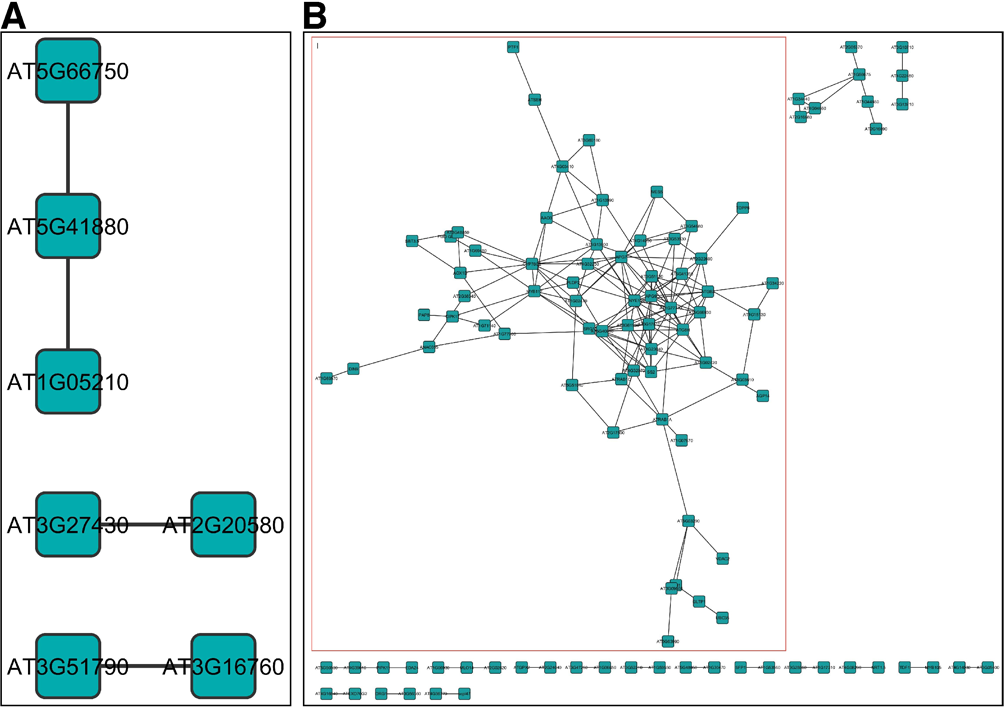

Supplement: Additional file 13: Figure S3. — Network analysis using genes that were up-regulated specifically in response to full length AC2. Sub-networks (red boxes) containing highly connected genes that were up-regulated in response to SCTV C2 and antisense SnRK1.2 at one (A) or two (B) dpi. [file 12870_2014_302_MOESM13_ESM.tiff]

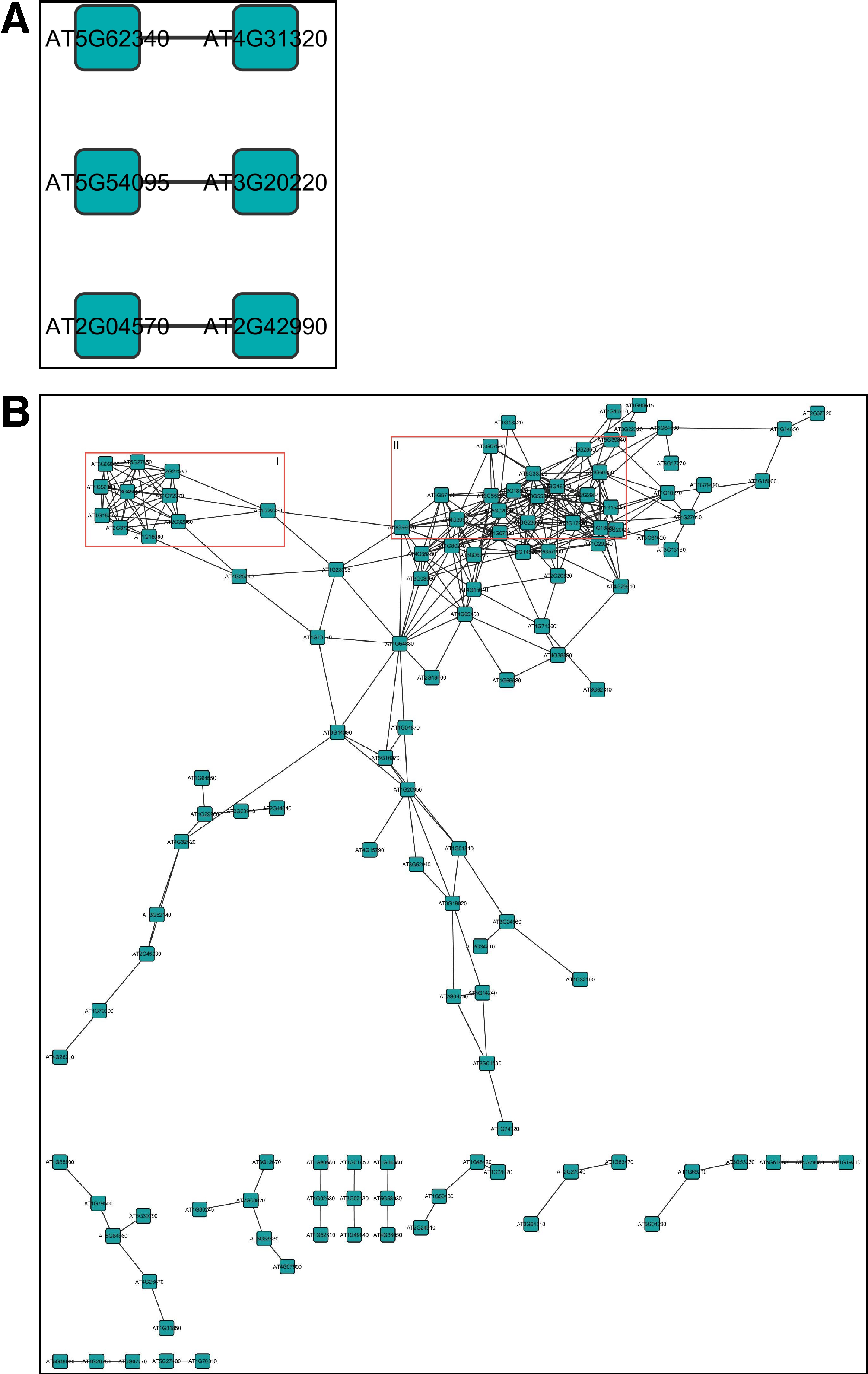

Supplement: Additional file 14: Figure S4. — Network analysis using genes that were down-regulated specifically in response to full length AC2. Sub-networks (red boxes) containing highly connected genes that were up-regulated in response to SCTV C2 and antisense SnRK1.2 at one (A) or two (B) dpi. [file 12870_2014_302_MOESM14_ESM.tiff]
